# Testosterone Supplementation: A Potential Therapeutic Strategy for Amyotrophic Lateral Sclerosis

**DOI:** 10.3390/biomedicines13030622

**Published:** 2025-03-04

**Authors:** Wenzhi Yang, Wendi Xiao, Xiangyi Liu, Hui Li, Tao Huang, Dongsheng Fan

**Affiliations:** 1 Department of Neurology, Peking University Third Hospital, Beijing 100080, China; 2111110464@bjmu.edu.cn (W.Y.); liuxiangyi@bjmu.edu.cn (X.L.); 2 Beijing Municipal Key Laboratory of Biomarker and Translational Research in Neurodegenerative Diseases, Beijing 100080, China; 3 School of Public Health, Peking University, Beijing 100080, China; 4 Key Laboratory of Carcinogenesis and Translational Research, Peking University, Beijing 100080, China

**Keywords:** amyotrophic lateral sclerosis, ALS, proteomics, mendelian randomization, MR, SHBG, testosterone, bioavailable testosterone

## Abstract

**Objectives:** Amyotrophic lateral sclerosis (ALS) is a progressive and fatal disease characterized by the degeneration of spinal cord and brain neurons. Proteomics combined with Mendelian randomization (MR) is an effective method for finding disease treatment targets. **Methods:** We aimed to seek new therapeutic targets for ALS. A large-scale GWAS on proteomics (4907 circulatory protein) with 35,559 individuals was included as the exposure data; a GWAS with 138,086 ALS patients was used as the outcome data; we found that a high level of sex hormone-binding globulin (SHBG) is a risk factor by MR analysis. Colocalization analyses were used to validate the causality between SHBG and ALS further. Functional enrichment found a high level of SHBG was associated with a low level of bioavailable testosterone. Two-sample MR confirmed the association of SHBG (400,210 samples), bioavailable testosterone (367,289 samples), and ALS. **Results**: A high level of SHBG, and a low level of bioavailable testosterone are risk factors for ALS. **Conclusions:** A low level of bioavailable testosterone is a risk factor for ALS. Although our study is relatively limited and cannot fully confirm that testosterone supplementation has a therapeutic effect on ALS, it offers a promising direction for ALS therapy.

## 1. Introduction

Amyotrophic lateral sclerosis (ALS) is a progressive and fatal disease characterized by the degeneration of spinal cord and brain neurons. In ALS, both upper and lower motor neurons are affected, leading to progressive motor symptoms, including muscle weakness, muscle atrophy, and spasms. The development of symptoms is irreversible, and most patients die of respiratory failure within 5 years. The pathogenesis of ALS is multifactorial and not completely understood. Approximately 10% of patients with ALS have a family history and are caused by certain genetic mutations, which are called familial ALS (fALS). The remaining 90% are sporadic ALS (sALS), patients with sALS have no specific pathogeny, and the possible causes could be physical damage, chemical toxicity, infection, or rare gene variants. So far, there are only limited treatment methods for ALS, there is an urgent need to develop new therapies for ALS [1,2,3].

Mendelian randomization (MR) analysis has been widely used in drug target development and drug reuse; the evaluation of historical drug development data showed that target-indication pairs with MR and colocalization support are more likely to be approved, which proves the value of this method in identifying potential therapeutic targets [4]. MR is a method that uses genetic variation to study the causal relationship between exposure and outcomes and relies on genome-wide association studies (GWASs). MR needs to meet the following three assumptions: (1) genetic variation is related to exposure; (2) genetic variation is independent of confounding factors between exposure and outcome; (3) genetic variation only affects outcomes through exposure [5]. Through proteomics, we can obtain the expression information of many proteins in diseases. Protein quantitative trait loci (pQTL) refers to SNP information related to protein expression levels. Through MR analysis between pQTL and the disease, we can find the proteins that are potential targets for disease therapy [6].

Through MR analysis between proteomics and ALS, we found that a high level of sex hormone-binding globulin (SHBG) is a risk factor for ALS. SHBG can bind testosterone and regulate the level of bioavailable testosterone. Most of the testosterone in the human body binds to SHBG, is known as SHBG-bound testosterone, and cannot be directly utilized by the body. Albumin-bound testosterone and free testosterone, also known as bioavailable testosterone, can be directly utilized by the human body. The binding between SHBG and testosterone is very stable, and a high level of SHBG reflects a low level of bioavailable testosterone [7]. A previous study found that the levels of SHBG in ALS patients were lower than those in non-ALS patients [8], suggesting that SHBG levels may be associated with the occurrence of ALS. However, there is no previous research demonstrating a causal relationship between bioavailable testosterone levels and ALS.

In this study, we conducted an MR analysis between proteomics and ALS and found that a high level of SHBG is a risk factor for ALS. Then, we conducted MR for SHBG and bioavailable testosterone in males and females, respectively, and MR between bioavailable testosterone and ALS in males and females, respectively. Our study found that a low level of bioavailable testosterone is a risk factor for ALS. Although this study cannot fully confirm the therapeutic effect of testosterone for ALS, it enlightened us to the notion that testosterone treatment may have potential in ALS therapy.

## 2. Materials and Methods

### 2.1. Study Design

**Figure 1:**
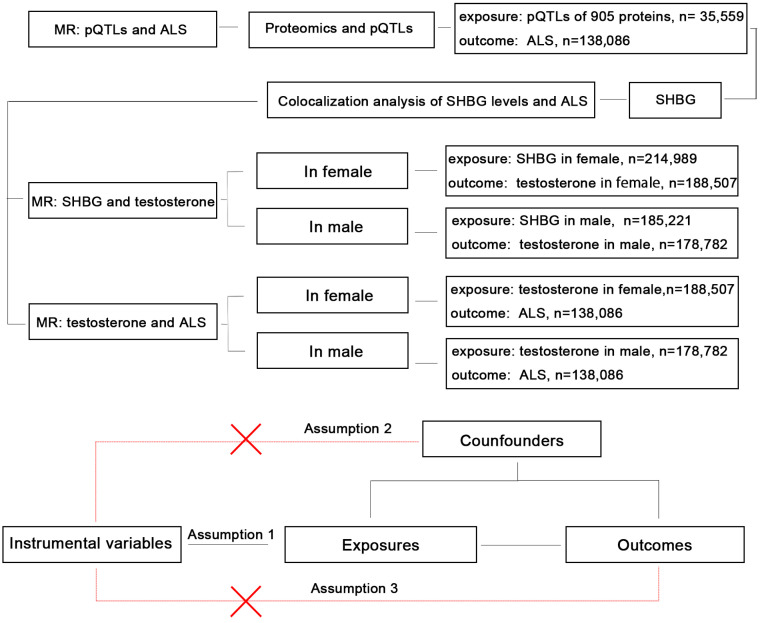
The flow chart of the study design.

The design of our study is presented in the flow chart (Figure 1). Our study included publicly available data from a large-scale GWAS on proteomics and five GWAS datasets from five traits. Firstly, we conducted an MR analysis between the pQTLs of 905 circulatory proteins and ALS; functional enrichment was used to identify the biological function of the significant proteins; colocalization analysis was used to verify the association between the most significant protein (SHBG) and ALS further. Then, to verify the association between SHBG, bioavailable testosterone, and ALS, we conducted four MR: (1) For SHBG and bioavailable testosterone levels in females. (2) For SHBG and bioavailable testosterone levels in males. (3) For bioavailable testosterone levels and ALS in females. (4) For bioavailable testosterone levels and ALS in males.

### 2.2. Proteomics and pQTL Data Sources

Data preprocessing for proteomics and pQTLs was conducted in R version 4.3.2. Summary-level statistics for genetic associations with 4907 circulatory protein levels were extracted from a large-scale QTL study [9], which included 35,559 European individuals. Proteomic analysis was performed using multiple and modified aptamer-based binding analysis (Soma Scan version 4). The protein levels were rank-inverse normal transformed according to age and sex; the rank inverse normal transformation was used to normalize the residuals, and the standardized value was used as the phenotype of genome-wide association analysis, using the BOLT-LMM linear mixed model. The original data of the GWAS can be obtained in the original study [9]. The genetic instruments were selected based on the criteria: (1) A *p* value < 5 × 10^−8^. (2) A cis-pQTL (pQTL within ±100 kb from the coding gene). According to the criteria, the pQTLs of 905 proteins were included in the next analysis.

### 2.3. GWAS Data Sources

All the GWAS data can be obtained at Open IEU GWAS Project (https://gwas.mrcieu.ac.uk, accessed on 1 June 2024). The accession number and detailed information are listed (Table 1).

SNPs used to predict SHBG levels are from the public summary statistics provided by Ruth et al. [10]; SHBG levels (nmol/L) were measured by one-step competitive analysis and two-step sandwich immunoassay on a Beckman Coulter unicel DXL 800 (Brea, CA, USA). SNPs that predict bioavailable testosterone levels are from the public summary statistics provided by Rebecca et al. [11]; the bioavailable testosterone levels were calculated by total testosterone and albumin (measured by BCG analysis on a Beckman Coulter AU5800, g/L) according to the Vermeulen equation (nmol/L) [13,14]. The summary statistical data of both the SHBG and bioavailable testosterone are from UK biobank data, which includes phenotypic and biological samples collected from about 500,000 individuals across Great Britain [15]. The ALS GWAS includes 29,612 patients with ALS and 122,656 controls [12]; all ALS patients were diagnosed and identified through a dedicated motor neuron disease clinic and were diagnosed by a neurologist specializing in motor neuron disease according to the revised El Escorial Criteria [16]. This is the largest GWAS study of ALS to date, and consists of 117 ALS cohorts from Europe.

### 2.4. MR Analysis

The MR analysis, heterogeneity test, pleiotropy test, and other sensitivity analyses were conducted in R (version 4.3.2) using the TwosampleMR package [17,18].

We performed MR analysis between proteomics and ALS based on the SNPs of circulatory proteins to find the relationships between circulating proteins and the risk of ALS; 905 proteins were included as the exposure factors, the threshold for instrumental SNPs selection was *p* < 5 × 10^−8^, the significant cutoff of *p* value in inverse variance weighted (IVW) method was adjusted by Bonferroni method (0.05/905 = 5.5249 × 10^−5^), the odd ratios (ORs) were calculated by the formula: OR = EXP(β), and the 95% confidence interval (CI) was calculated by the formula [EXP(β − 1.96 × SE), EXP(β + 1.96 × SE)].

For the MR analysis of SHBG, bioavailable testosterone, and ALS, the threshold for instrumental SNP selection was *p* < 5 × 10^−8^; five methods, including MR Egger, weighted median, IVW, simple mode, and weighted mode, were used for the MR analysis, and the significant cutoff of *P* value was set as *p* < 0.05.

### 2.5. Functional Enrichment Analysis

Protein functional enrichments were conducted in the DisGeNET [19] category by Metascape [20]. All genes in the genome were used as the enrichment background. Terms with a *p* < 0.01, a minimum count of 3, and an enrichment factor > 1.5 were collected and grouped into clusters based on their membership similarities.

### 2.6. Colocalization Analysis

In the colocalization analysis, the colocalization area was set as ±100 kb from the gene’s genome position. For each locus, the Bayesian method evaluated the support of the following five exclusion hypotheses: H0—the locus is not associated with two traits; H1—the locus is only associated with trait 1; H2—the locus is only associated with trait 2; H3—the locus is associated with both traits, but the causal variants for the two traits are different; H4—the locus is associated with both traits, and the two traits share a causal variant. We set the prior probability that SNP is only related to trait 1 (P1) as 10^−4^; the probability that SNP was only associated with trait 2 (P2) was 10^−4^; and the probability of SNP being associated with both traits (P12) was set as 10^−5^. The a posteriori probability for each hypothesis, PP.H0, PP.H1, PP.H2, PP.H3, and PP.H4, was calculated and PP.H4 > 0.75 was defined as strong colocalization. The lead SNP is highlighted in the figure. The colocalization analysis was conducted using the R (version 4.3.2) package coloc [21,22,23]. The visualization of the colocalization analysis was conducted using the R package locuscomparer [24].

### 2.7. Manhattan Plot

Manhattan plots of SHBG levels (pQTL) and ALS (GWAS) were drawn using the R (version 4.3.2) package CMplot. The pQTL of SHBG levels contained 16,582,620 SNPs, the GWAS of ALS contained 10,461,755 SNPs, and 9,031,523 SNPs remained after merging.

## 3. Results

### 3.1. MR Analysis Between Proteomics and ALS

**Figure 2:**
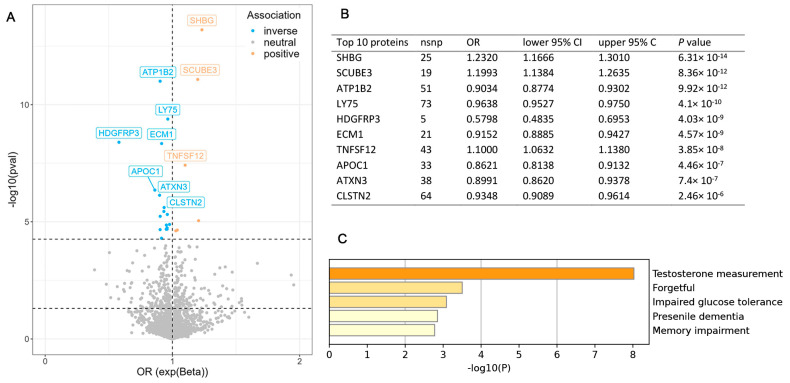
The results of MR analysis between proteomics and ALS, and the proteins’ functional enrichment. (**A**) The results of MR. (**B**) The information on the top 10 proteins. (**C**) The proteins’ functional enrichment.

We conducted MR analysis between pQTLs of 905 proteins and ALS, the cutoff of *P* value was adjusted by the Bonferroni method (0.05/905 = 5.5249 × 10^−5^), and 21 significant proteins were found (SHBG, SCUBE3, ATP1B2, LY75, HDGFRP3, ECM1, TNFSF12, APOC1, ATXN3, CLSTN2, NT5C3L, TIMD4, NID1, TCN2, LCT, IL1RL1, SERPING1, UGT1A6, HGFAC, PLA2R1, DECR2) (Figure 2A). The specific *P* value and OR of the top 10 proteins (Figure 2B) and the proteins’ functional enrichment analysis (Figure 2C) are listed. The detailed information on all 21 proteins is shown in the Supplementary Materials (Table S1), and the functional enrichment of all 21 proteins is shown in the Supplementary Materials (Figure S1). A high level of three proteins (SHBG, SCUEB3 and TNFSF22) and a low level of seven proteins (ATP1B2, LY75, HDGFRP3, ECM2, APOC1, ATXN3, CLSTN2) are risk factors for ALS. Protein functional enrichment shows the most significant function is the testosterone measurement (*p* = 6.30968 × 10^−9^). SHBG is the most significant protein among the results, with an OR = 1.2320 and 95% CI = (1.1666, 1.3010). Considering that SHBG can affect the level of bioavailable testosterone, we chose SHBG as the next research target.

### 3.2. Colocalization Analysis of SHBG and ALS

**Figure 3:**
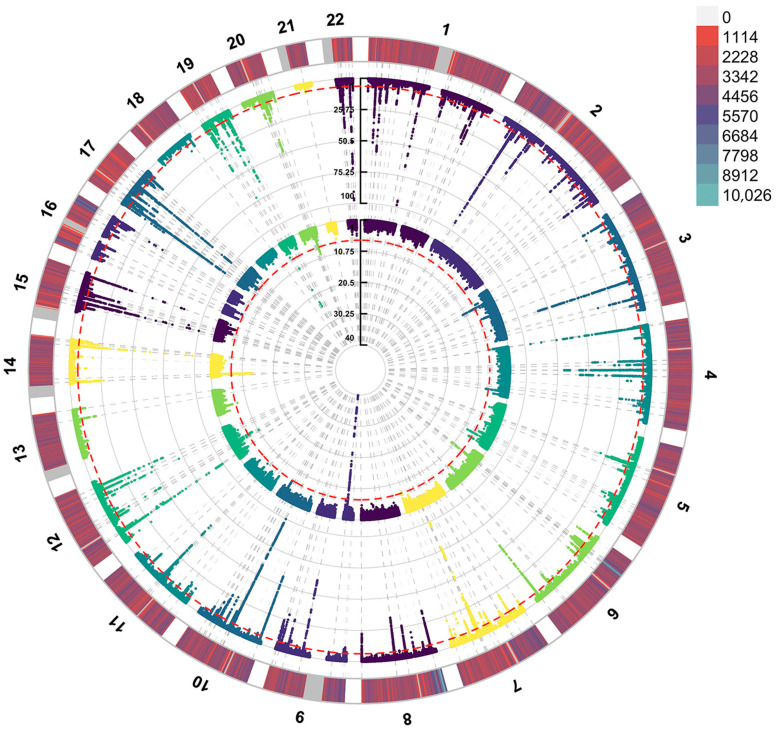
The Manhattan plot of SHBG levels and ALS. Outer circle: SHBG levels. Inner circle: ALS. Red dotted line showed the significant correlation.

**Figure 4:**
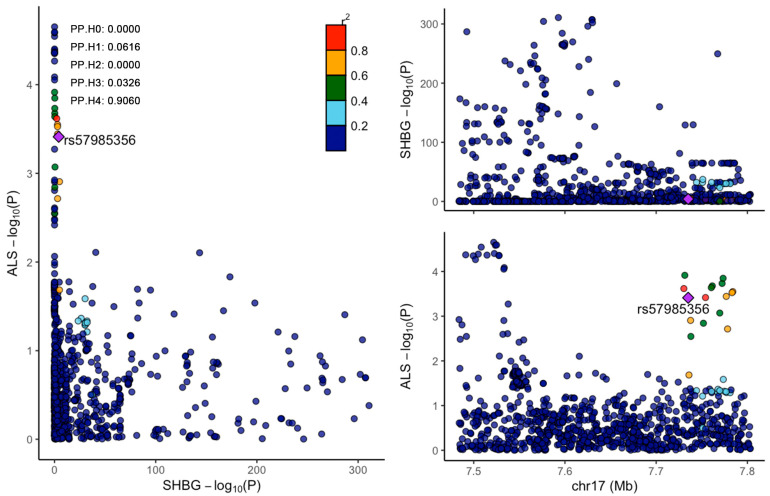
The results of colocalization of SHBG levels and ALS. H4 = 0.9060, the lead SNP is rs57985356. Purple symbol: lead SNP.

We conducted colocalization analyses of SHBG and ALS. Manhattan maps were drawn to observe the distribution of SNPs related to the SHBG level and ALS (Figure 3). The SHBG gene is located at chromosome 17: 7583647–7703502; we selected 7483647–7803532 (±100 kb from SHBG position) to conduct colocalization. The results show that SHBG and ALS shared the causal variants PP.H4 = 0.9060 (Figure 4).

### 3.3. MR Analysis Between SHBG and Bioavailable Testosterone in Males and Females

**Figure 5:**
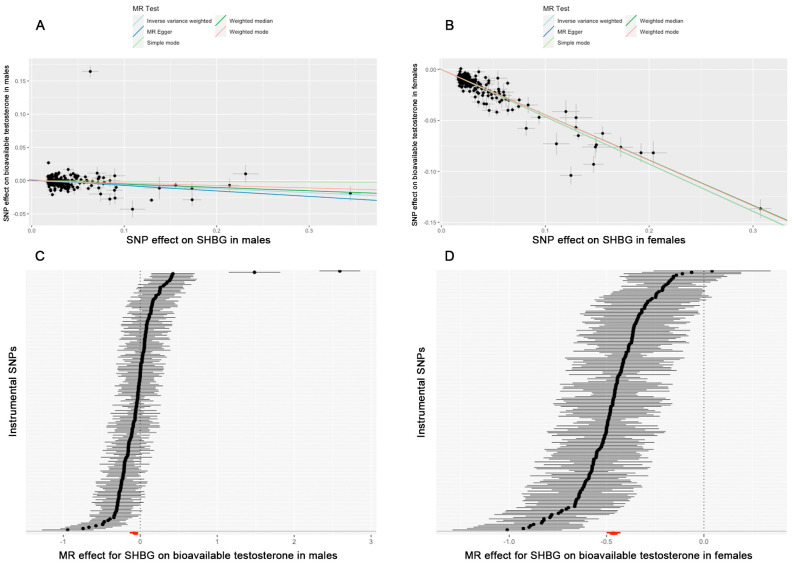
The results of MR between SHBG and bioavailable testosterone. (**A**) A scatter plot of MR in males. (**B**) A scatter plot of MR in females. (**C**) The effects of instrumental SNPs of MR in males. (**D**) The effects of instrumental SNPs of MR in females.

In total, 158 SNPs in males and 145 SNPs in females were selected as instrumental variables. The MR between SHBG and testosterone shows that a high level of SHBG is the cause of a low level of bioavailable testosterone in both males (*p* = 0.0001) and females (*p* = 0.0000), but the effect is stronger in females (OR = 0.6291 in females and OR = 0.9415 in males) (Figure 5). The heterogeneity test showed the heterogeneity is significant between instrumental SNPs in both males and females (Q_p = 0.0000 in females, Q_p = 0.0000 in males); therefore, we chose the random effects model to conduct the MR analysis. The pleiotropy test shows there is no pleiotropy between instrumental SNPs (*p* = 0.34776 in males, *p* = 0.9448 in females) (Supplementary Materials Table S2). The sensitive analysis including a leave-one-out and funnel plot are shown in the Supplementary Materials (Figures S2–S5).

### 3.4. MR Analysis Between Bioavailable Testosterone and ALS in Males and Females

**Figure 6:**
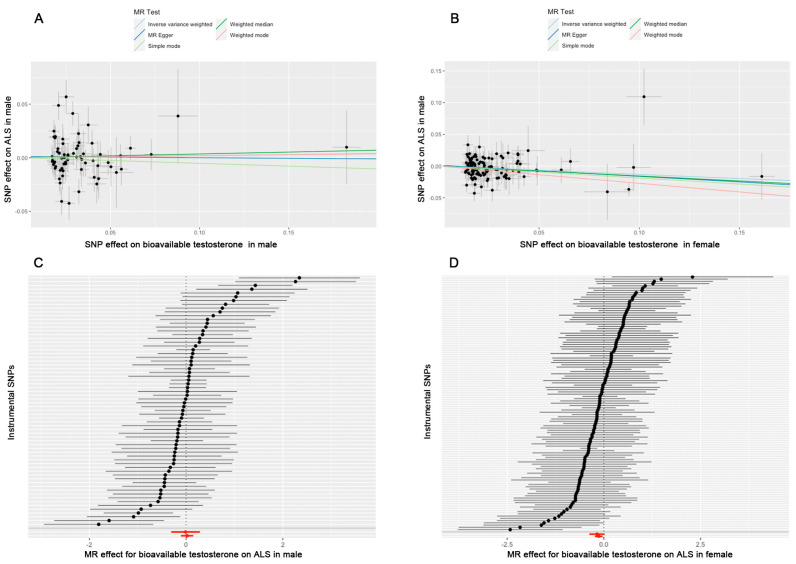
The results of MR between bioavailable testosterone and ALS. (**A**) A scatter plot of MR in males. (**B**) A scatter plot of MR in females. (**C**) The effects of instrumental SNPs of MR in males. (**D**) The effects of instrumental SNPs of MR in females.

To evaluate the effect of bioavailable testosterone in ALS, we conducted an MR analysis between bioavailable testosterone and ALS in males and females. In males, there is no significant causal relationship between bioavailable testosterone and ALS (*p* = 0.7548). In females, the causal relationship between bioavailable testosterone and ALS is significant (*p* = 0.0098) (Figure 6). A low level of bioavailable testosterone is a risk factor for ALS, with an OR = 0.8768 and a 95% CI = (0.7934, 0.9687). This result is consistent with the MR between SHBG and ALS (a high level of SHBG is a risk factor for ALS, and a high level of SHBG reflects a low level of bioavailable testosterone, especially in females). The heterogeneity test shows there is no significant heterogeneity between instrumental SNPs (Q_p = 0.1137). The pleiotropy test shows there is no pleiotropy between instrumental SNPs (*p* = 0.6080) (Supplementary Materials, Table S3). The sensitivity analysis, including the leave-one-out and funnel plots, shows that this result is robust and without significant bias; the figures are listed in the Supplementary Materials (Figures S6–S9).

## 4. Discussion

High-throughput proteomic analysis of population biobanks may accelerate our understanding of diseases. In our study, we found that a high level of SHBG is a risk factor for ALS through proteomics and pQTL. Considering the SHBG level is related to bioavailable testosterone levels, we conducted an MR analysis between bioavailable testosterone and ALS and found that low bioavailable testosterone is a risk factor for ALS in females. Our study reports the causality between bioavailable testosterone and ALS.

SHBG is a plasma sex hormone-binding protein secreted by the liver, which can prevent testosterone from binding to the intracellular androgen receptor. Evidence suggests that the plasma SHBG level may be an effective biomarker of neurodegenerative diseases [25,26]. One study reported that a high level of SHBG is associated with an increased risk of ALS [8], which is consistent with our study. SHBG was also studied in Parkinson’s disease, and a previous study revealed that there is no difference in SHBG levels between Parkinson’s disease patients and healthy subjects [27]. In general, there are few studies on SHBG and neurodegenerative diseases, and more studies are needed to explain the relationship between SHBG and neurodegenerative diseases.

Testosterone is the most important androgen in the human body, mainly synthesized by testicular interstitial cells, and can be secreted by the adrenal gland. In addition, female ovaries can also secrete androgens. The testosterone level in males is approximately 10 times higher than that in females. The main physiological function of testosterone is to promote the development and growth of reproductive organs, stimulate sexual desire, and at the same time, promote and maintain the development of male secondary sexual characteristics, maintain the function and spermatogenic effect of the prostate and seminal vesicles. Testosterone also plays an important nutritional role in neurons [28,29,30]. Kennedy’s disease, a genetic disease caused by mutations in the androgen receptor gene on the X chromosome, has symptoms very similar to ALS, including progressive myasthenia and muscle atrophy [31]. Athletes who require a high degree of endurance have an increased susceptibility to ALS [32]. ALS risk increases in women with earlier menopause [33]. All the information above suggests testosterone deficiency may be related to ALS.

Previous studies have shown that testosterone is associated with ALS. A study with 37 ALS patients and 57 healthy subjects revealed that free testosterone was significantly decreased in the ALS patients, but no differences were observed in dehydroepiandrosterone sulphate, 17-betaestradiol, and total testosterone, which is consistent with our study [34]. Another study, with 13 ALS patients and 22 healthy subjects, found that free testosterone levels did not show any significant differences in ALS cerebrospinal fluid (CSF), but CSF dihydrotestosterone levels were significantly decreased in ALS patients [35]. Another interesting study (47 ALS patients and 63 healthy subjects) showed that patients with ALS have a lower ratio of index to ring finger length (2D:4D ratio); a low 2D:4D ratio is considered a surrogate marker for high prenatal testosterone levels in both men and women, and a high level of prenatal testosterone may lead to androgen insensitivity [36]. However, a study, which is based on a screen of medicare prescription drugs, found that using testosterone was related with a higher ALS risk in women (OR = 3.93), and this study also reported that tamoxifen, a selective estrogen receptor modulator, was related with a lower ALS risk [37].

Previous studies and our own study all found the sex-specific effect of testosterone level in ALS, and the mechanism of the disparity is still unclear. To explain this disparity, we proposed three hypotheses: (1) Testosterone level may be related with estrogen level. An experiment in rats model showed a potential synergism between androgen and estrogen [38]. And there is evidence showing that tamoxifen, a selective estrogen receptor modulator, is related with lower ALS risk; a previous study also reported the neuroprotective effect of estrogen. (2) Androgen receptor-mediated transcription plays an important role in both males and females [39]. In females, the gene expression mediated by the activation of androgen receptor may be different to that of males. (3) Compared with males, females have lower testosterone levels, but testosterone also has important biological functions in females [40], which suggests that females may be more sensitive to fluctuations in testosterone levels.

In previous research on ALS animal models, one study showed that testosterone treatment has a beneficial role in the symptom progress in the Wobbler mouse, which is an ALS model mouse [41]. Another study showed that androgen receptor antagonists accelerate the onset of the SOD1-G93A mouse model, leading to pathological muscle deterioration [42]. However, there are studies with the opposite conclusions. One study showed that androgen ablation by surgical castration extends the survival duration of the SOD1-G93A mouse model, and nandrolone decanoate (a kind of testosterone medicine) treatment worsened disease manifestations of the hSOD1-G93A mouse model [43]. So far, there is no clinical trial of androgen replacement therapy for ALS patients.

Although previous studies and our study have established the association between testosterone and ALS, there has been no research on the mechanism of testosterone’s effect on ALS. As a kind of androgen, testosterone can improve motor ability through various pathways, including neuroprotection, anti-inflammatory effects, and muscle maintenance. But the impair of these functions caused by low testosterone levels is not likely to lead to irreversible devastating diseases like ALS. We believe the mechanism of testosterone’s effect on ALS exists at a more fundamental level. It is known that androgens play and important role in reproduction, and the reproductive function of androgens is mediated by various physiological processes from brain function to specific cell proliferation and apoptosis [44]. The functions of androgens rely on androgen receptors. Unliganded androgen receptors exist in the cytoplasm and, when combined with androgen, the androgen receptor will be transported to the nucleus and may mediate the transcription of various genes. We know that several ALS-related proteins, including TDP-43, SOD1 and FUS, are RNA-binding proteins which can mediate the expression of genes [45,46,47], in a way that is similar to androgen receptors. In particular, TDP-43—the nucleocytoplasmic transport defects of TDP-43 have been confirmed in ALS patients. With this in mind, we hypothesize that the mechanism of testosterone’s effect on ALS is the mediating of gene expression by combining with androgen receptors. Low testosterone levels or an insensitivity of androgen receptors will lead to function loss in important genes or the disruption of RNA homeostasis, which will lead to the apoptosis of neurons. To clarify this issue, we need to study the changes in gene expression levels under conditions of low testosterone levels and low androgen receptor levels, and search for factors related to ALS in the changes in gene expression.

There are several limitations in our study. For proteomics, we only included the pQTLs located at or close to the gene of origin (±100 kb from each gene’s genome position); the pQTLs far from the original gene were not included, which may lose some information on the genetic association between pQTLs and proteins. We only analyzed the relationship between bioavailable testosterone levels and ALS; however, testosterone insensitivity may cause symptoms like low levels of bioavailable testosterone, which cannot be evaluated in MR analysis. In addition, the results of the MR analyses should be interpreted with caution. Although there is a *p* > 0.05 between bioavailable testosterone and ALS in males, it cannot be confirmed that there is no association between them. Although we found the relationship between testosterone and ALS through MR, the relationship needs to be verified by further animal or clinical study. Despite the limitations of our study, the results of this study enlighten us regarding the notion that testosterone supplementation may be a promising direction for ALS therapy.

## 5. Conclusions

Our study found that a low level of bioavailable testosterone is a risk factor for ALS. Although this study cannot fully confirm the therapeutic effect of testosterone for ALS, it enlightened us to the notion that testosterone treatment may have potential in ALS therapy.

## Figures and Tables

**Table 1:** The information of included GWAS.

Trait	Accession Number	Population	Sample Size	Year	Reference
SHBG levels in females	ieu-b-4870	European	214,989	2022	Rebecca Richmond [10]
SHBG levels in males	sieu-b-4871	European	185,221	2022	Rebecca Richmond [10]
Bioavailable testosterone levels in females	ebi-a-GCST90012102	European	188,507	2020	Ruth KS [11]
Bioavailable testosterone levels in males	ebi-a-GCST90012103	European	178,782	2020	Ruth KS [11]
ALS	ebi-a-GCST90027163	European	138,086	2021	van Rheenen W [12]

## Data Availability

The data on pQTL and GWAS are described in the Methods section, and all other data analyzed in this study can be obtained upon reasonable request to the authors.

## Supplementary Materials

The following supporting information can be downloaded at: https://www.mdpi.com/article/10.3390/biomedicines13030622/s1. Table S1: The information of 21 significant proteins. Table S2: The results of MR between SHBG and bioavailable testosterone in males and females. Table S3: The results of MR between bioavailable testosterone and ALS in males and females. Figure S1: Functional enrichment of 21 proteins. Figure S2: Leave-one-out (SHBG and bioavailable testosterone in male). Figure S3: Leave-one-out (SHBG and bioavailable testosterone in female). Figure S4: Funnel plot (SHBG and bioavailable testosterone in male). Figure S5: Funnel plot (SHBG and bioavailable testosterone in female). Figure S6: Leave-one-out (Bioavailable testosterone and ALS in male). Figure S7: Leave-one-out (Bioavailable testosterone and ALS in female). Figure S8: Funnel plot (Bioavailable testosterone and ALS in male). Figure S9: Funnel plot (Bioavailable testosterone and ALS in female).

## References

[1] Tzeplaeff L., Wilfling S., Requardt M.V., Herdick M. (2023). Current State and Future Directions in the Therapy of ALS. Cells.

[2] Ilieva H., Vullaganti M., Kwan J. (2023). Advances in molecular pathology, diagnosis, and treatment of amyotrophic lateral sclerosis. BMJ.

[3] Khamaysa M., Pradat P.F. (2022). Status of ALS Treatment, Insights into Therapeutic Challenges and Dilemmas. J. Pers. Med..

[4] Zheng J., Haberland V., Baird D., Walker V., Haycock P.C., Hurle M.R., Gutteridge A., Erola P., Liu Y., Luo S. (2020). Phenome-wide Mendelian randomization mapping the influence of the plasma proteome on complex diseases. Nat. Genet..

[5] Birney E. (2022). Mendelian Randomization. Cold Spring Harb. Perspect. Med..

[6] Molendijk J., Parker B.L. (2021). Proteome-wide Systems Genetics to Identify Functional Regulators of Complex Traits. Cell Syst..

[7] Hammond G.L. (2016). Plasma steroid-binding proteins: Primary gatekeepers of steroid hormone action. J. Endocrinol..

[8] Ou Y.N., Yang L., Wu B.S., Tan L., Yu J.T. (2022). Causal effects of serum sex hormone binding protein levels on the risk of amyotrophic lateral sclerosis: A mendelian randomization study. Ann. Transl. Med..

[9] Ferkingstad E., Sulem P., Atlason B.A., Sveinbjornsson G., Magnusson M.I., Styrmisdottir E.L., Gunnarsdottir K., Helgason A., Oddsson A., Halldorsson B.V. (2021). Large-scale integration of the plasma proteome with genetics and disease. Nat. Genet..

[10] Nounu A., Kar S.P., Relton C.L., Richmond R.C. (2022). Sex steroid hormones and risk of breast cancer: A two-sample Mendelian randomization study. Breast Cancer Res..

[11] Ruth K.S., Day F.R., Tyrrell J., Thompson D.J., Wood A.R., Mahajan A., Beaumont R.N., Wittemans L., Martin S., Busch A.S. (2020). Using human genetics to understand the disease impacts of testosterone in men and women. Nat. Med..

[12] van Rheenen W., van der Spek R.A.A., Bakker M.K., van Vugt J., Hop P.J., Zwamborn R.A.J., de Klein N., Westra H.J., Bakker O.B., Deelen P. (2021). Common and rare variant association analyses in amyotrophic lateral sclerosis identify 15 risk loci with distinct genetic architectures and neuron-specific biology. Nat. Genet..

[13] Vermeulen A., Verdonck L., Kaufman J.M. (1999). A critical evaluation of simple methods for the estimation of free testosterone in serum. J. Clin. Endocrinol. Metab..

[14] Chung M.C., Gombar S., Shi R.Z. (2017). Implementation of Automated Calculation of Free and Bioavailable Testosterone in Epic Beaker Laboratory Information System. J. Pathol. Inform..

[15] Bycroft C., Freeman C., Petkova D., Band G., Elliott L.T., Sharp K., Motyer A., Vukcevic D., Delaneau O., O’Connell J. (2018). The UK Biobank resource with deep phenotyping and genomic data. Nature.

[16] Brooks B.R., Miller R.G., Swash M., Munsat T.L. (2000). El Escorial revisited: Revised criteria for the diagnosis of amyotrophic lateral sclerosis. Amyotroph. Lateral Scler. Other Motor Neuron Disord..

[17] Hemani G., Zheng J., Elsworth B., Wade K.H., Haberland V., Baird D., Laurin C., Burgess S., Bowden J., Langdon R. (2018). The MR-Base platform supports systematic causal inference across the human phenome. eLife.

[18] Lawlor D.A. (2016). Commentary: Two-sample Mendelian randomization: Opportunities and challenges. Int. J. Epidemiol..

[19] Pinero J., Bravo A., Queralt-Rosinach N., Gutierrez-Sacristan A., Deu-Pons J., Centeno E., Garcia-Garcia J., Sanz F., Furlong L.I. (2017). DisGeNET: A comprehensive platform integrating information on human disease-associated genes and variants. Nucleic Acids Res..

[20] Zhou Y., Zhou B., Pache L., Chang M., Khodabakhshi A.H., Tanaseichuk O., Benner C., Chanda S.K. (2019). Metascape provides a biologist-oriented resource for the analysis of systems-level datasets. Nat. Commun..

[21] Giambartolomei C., Vukcevic D., Schadt E.E., Franke L., Hingorani A.D., Wallace C., Plagnol V. (2014). Bayesian test for colocalisation between pairs of genetic association studies using summary statistics. PLoS Genet..

[22] Wallace C. (2020). Eliciting priors and relaxing the single causal variant assumption in colocalisation analyses. PLoS Genet..

[23] Wallace C. (2021). A more accurate method for colocalisation analysis allowing for multiple causal variants. PLoS Genet..

[24] Liu B., Gloudemans M.J., Rao A.S., Ingelsson E., Montgomery S.B. (2019). Abundant associations with gene expression complicate GWAS follow-up. Nat. Genet..

[25] Muller M., Schupf N., Manly J.J., Mayeux R., Luchsinger J.A. (2010). Sex hormone binding globulin and incident Alzheimer’s disease in elderly men and women. Neurobiol. Aging.

[26] Xu W., Su B.J., Shen X.N., Bi Y.L., Tan C.C., Li J.Q., Cao X.P., Dong Q., Tan L., Alzheimer’s Disease Neuroimaging I. (2020). Plasma sex hormone-binding globulin predicts neurodegeneration and clinical progression in prodromal Alzheimer’s disease. Aging.

[27] Nitkowska M., Tomasiuk R., Czyzyk M., Friedman A. (2015). Prolactin and sex hormones levels in males with Parkinson’s disease. Acta Neurol. Scand..

[28] Bianchi V.E., Rizzi L., Bresciani E., Omeljaniuk R.J., Torsello A. (2020). Androgen Therapy in Neurodegenerative Diseases. J. Endocr. Soc..

[29] Carteri R.B., Kopczynski A., Rodolphi M.S., Strogulski N.R., Sartor M., Feldmann M., De Bastiani M.A., Duval Wannmacher C.M., de Franceschi I.D., Hansel G. (2019). Testosterone Administration after Traumatic Brain Injury Reduces Mitochondrial Dysfunction and Neurodegeneration. J. Neurotrauma.

[30] Gurer B., Kertmen H., Kasim E., Yilmaz E.R., Kanat B.H., Sargon M.F., Arikok A.T., Erguder B.I., Sekerci Z. (2015). Neuroprotective effects of testosterone on ischemia/reperfusion injury of the rabbit spinal cord. Injury.

[31] Breza M., Koutsis G. (2019). Kennedy’s disease (spinal and bulbar muscular atrophy): A clinically oriented review of a rare disease. J. Neurol..

[32] Stipa G., Ancidoni A., Vanacore N., Bellomo G. (2023). Raw Water and ALS: A Unifying Hypothesis for the Environmental Agents Involved in ALS. Ann. Neurosci..

[33] Raymond J., Mehta P., Larson T., Pioro E.P., Horton D.K. (2021). Reproductive History and Age of Onset for Women Diagnosed with Amyotrophic Lateral Sclerosis: Data from the National ALS Registry: 2010–2018. Neuroepidemiology.

[34] Militello A., Vitello G., Lunetta C., Toscano A., Maiorana G., Piccoli T., La Bella V. (2002). The serum level of free testosterone is reduced in amyotrophic lateral sclerosis. J. Neurol. Sci..

[35] Sawal N., Kaur J., Kaur K., Gombar S. (2020). Dihydrotestosterone in Amyotrophic lateral sclerosis-The missing link?. Brain Behav..

[36] Vivekananda U., Manjalay Z.R., Ganesalingam J., Simms J., Shaw C.E., Leigh P.N., Turner M.R., Al-Chalabi A. (2011). Low index-to-ring finger length ratio in sporadic ALS supports prenatally defined motor neuronal vulnerability. J. Neurol. Neurosurg. Psychiatry.

[37] Pfeiffer R.M., Mayer B., Kuncl R.W., Check D.P., Cahoon E.K., Rivera D.R., Freedman D.M. (2020). Identifying potential targets for prevention and treatment of amyotrophic lateral sclerosis based on a screen of medicare prescription drugs. Amyotroph. Lateral Scler. Front. Degener..

[38] Handa R.J., Stadelman H.L., Resko J.A. (1987). Effect of estrogen on androgen receptor dynamics in female rat pituitary. Endocrinology.

[39] Altintas U.B., Seo J.H., Giambartolomei C., Ozturan D., Fortunato B.J., Nelson G.M., Goldman S.R., Adelman K., Hach F., Freedman M.L. (2024). Decoding the epigenetics and chromatin loop dynamics of androgen receptor-mediated transcription. Nat. Commun..

[40] McHenry J., Carrier N., Hull E., Kabbaj M. (2014). Sex differences in anxiety and depression: Role of testosterone. Front. Neuroendocrinol..

[41] Lara A., Esperante I., Meyer M., Liere P., Di Giorgio N., Schumacher M., Guennoun R., Gargiulo-Monachelli G., De Nicola A.F., Gonzalez Deniselle M.C. (2021). Neuroprotective Effects of Testosterone in Male Wobbler Mouse, a Model of Amyotrophic Lateral Sclerosis. Mol. Neurobiol..

[42] McLeod V.M., Lau C.L., Chiam M.D.F., Rupasinghe T.W., Roessner U., Djouma E., Boon W.C., Turner B.J. (2019). Androgen receptor antagonism accelerates disease onset in the SOD1(G93A) mouse model of amyotrophic lateral sclerosis. Br. J. Pharmacol..

[43] Aggarwal T., Polanco M.J., Scaramuzzino C., Rocchi A., Milioto C., Emionite L., Ognio E., Sambataro F., Galbiati M., Poletti A. (2014). Androgens affect muscle, motor neuron, and survival in a mouse model of SOD1-related amyotrophic lateral sclerosis. Neurobiol. Aging.

[44] Roy A.K., Tyagi R.K., Song C.S., Lavrovsky Y., Ahn S.C., Oh T.S., Chatterjee B. (2001). Androgen receptor: Structural domains and functional dynamics after ligand-receptor interaction. Ann. N. Y. Acad. Sci..

[45] Pham J., Keon M., Brennan S., Saksena N. (2020). Connecting RNA-Modifying Similarities of TDP-43, FUS, and SOD1 with MicroRNA Dysregulation Amidst A Renewed Network Perspective of Amyotrophic Lateral Sclerosis Proteinopathy. Int. J. Mol. Sci..

[46] Daigle J.G., Lanson N.A., Smith R.B., Casci I., Maltare A., Monaghan J., Nichols C.D., Kryndushkin D., Shewmaker F., Pandey U.B. (2013). RNA-binding ability of FUS regulates neurodegeneration, cytoplasmic mislocalization and incorporation into stress granules associated with FUS carrying ALS-linked mutations. Hum. Mol. Genet..

[47] Hallegger M., Chakrabarti A.M., Lee F.C.Y., Lee B.L., Amalietti A.G., Odeh H.M., Copley K.E., Rubien J.D., Portz B., Kuret K. (2021). TDP-43 condensation properties specify its RNA-binding and regulatory repertoire. Cell.

