# Supplementary material for: Testosterone Supplementation: A Potential Therapeutic Strategy for Amyotrophic Lateral Sclerosis
Source: Biomedicines. 2025 Mar 4;13(3):622. doi: 10.3390/biomedicines13030622 (PMC11940241; doi:10.3390/biomedicines13030622)
Supplement: Supplementary file 1 [file biomedicines-13-00622-s001.zip › biomedicines-3442731-supplementary.pdf]

Supplemental Materials

**Exposures**	**β**	***P* value**
**SHBG**	0.208600863	6.31E-14
**SCUBE3**	0.181757969	8.36E-12
**ATP1B2**	-0.101563691	9.92E-12
**LY75**	-0.036890465	4.10E-10
**HDGFRP3**	-0.545049803	4.03E-09
**ECM1**	-0.088635148	4.57E-09
**TNFSF12**	0.095273693	3.85E-08
**APOC1**	-0.14839407	4.46E-07
**ATXN3**	-0.106363128	7.40E-07
**CLSTN2**	-0.067444708	2.46E-06
**NT5C3L**	-0.069203413	3.62E-06
**TIMD4**	-0.100807911	5.86E-06
**NID1**	0.187060882	9.01E-06
**TCN2**	-0.022807713	1.30E-05
**LCT**	-0.048429567	1.39E-05
**IL1RL1**	-0.04279706	2.08E-05
**SERPING1**	-0.049909605	2.09E-05
**UGT1A6**	-0.101807776	2.17E-05
**HGFAC**	0.037723664	2.26E-05
**PLA2R1**	0.026586298	2.44E-05
**DECR2**	-0.089501186	5.07E-05

Table S1: The information of 21 significant proteins

	**Male**				
**MR analysis (197 SNPs)**	Method	OR	lower 95% CI	upper 95% CI	*P* value
	MR Egger	0.9208	0.8233	1.0298	0.0039
	Weighted median	0.9504	0.8685	1.0401	0.0002
	IVW	0.9415	0.8860	1.0005	0.0001
	Simple mode	0.9936	0.7687	1.2844	0.8603
	Weighted mode	0.9639	0.8582	1.0826	0.0580
**Heterogeneity test**	Method	Q	Q_df	Q_*P* value	
	MR Egger	1024.4800	188.0000	0.0000	
	IVW	1029.3140	189.0000	0.0000	
**Pleiotropy test**	Egger_intercept	SE	*P* value		
	0.0011	0.0011	0.3475		
	**Female**				
**MR analysis (200 SNPs)**	Method	OR	lower 95% CI	upper 95% CI	*P* value
	MR Egger	0.6285	0.5433	0.7270	0.0000
	Weighted median	0.6411	0.5747	0.7152	0.0000
	IVW	0.6291	0.5822	0.6798	0.0000
	Simple mode	0.6283	0.4732	0.8341	0.0000
	Weighted mode	0.6399	0.5362	0.7636	0.0000
**Heterogeneity test**	Method	Q	Q_df	Q_*P* value	
	MR Egger	471.7909	186.0000	0.0000	
	IVW	471.8031	187.0000	0.0000	
**Pleiotropy test**	Egger_intercept	SE	*P* value		
	0.0000	0.0007	0.9448		

Table S2: The results of MR between SHBG and bioavailable testosterone in males and females.

Male

	Method	OR	lower 95% CI	upper 95% CI	*P* value
**MR analysis (96 SNPs)**	MR Egger	0.9891	0.7346	1.3319	0.9429
	Weighted median	1.0357	0.8999	1.1920	0.6252
	IVW	1.0206	0.8980	1.1600	0.7548
	Simple mode	0.9489	0.7196	1.2513	0.7113
	Weighted mode	1.0188	0.8599	1.2070	0.8305
**Heterogeneity test**	Method	Q	Q_df	Q_*P* value	
	MR Egger	113.5626	64	0.0001	
	IVW	113.6556	64	0.0002	
**Pleiotropy test**	Egger_intercept	SE	*P* value		
	0.0011	0.0048	0.8197		

Female

	Method	OR	lower 95% CI	upper 95% CI	*P* value
**MR analysis (148 SNPs)**	MR Egger	0.8383	0.6878	1.0217	0.0833
	Weighted median	0.8546	0.7314	0.9987	0.0481
	IVW	0.8767	0.7934	0.9687	0.0098
	Simple mode	0.8273	0.5757	1.1887	0.3074
	Weighted mode	0.7614	0.6406	0.9050	0.0025
**Heterogeneity test**	Method	Q	Q_df	Q_*P* value	
	MR Egger	130.0145	111	0.1049	
	IVW	130.3245	112	0.1137	
**Pleiotropy test**	Egger_intercept	SE	*P* value		
	0.0014	0.0027	0.6080		

Table S3: The results of MR between bioavailable testosterone and ALS in males and females.

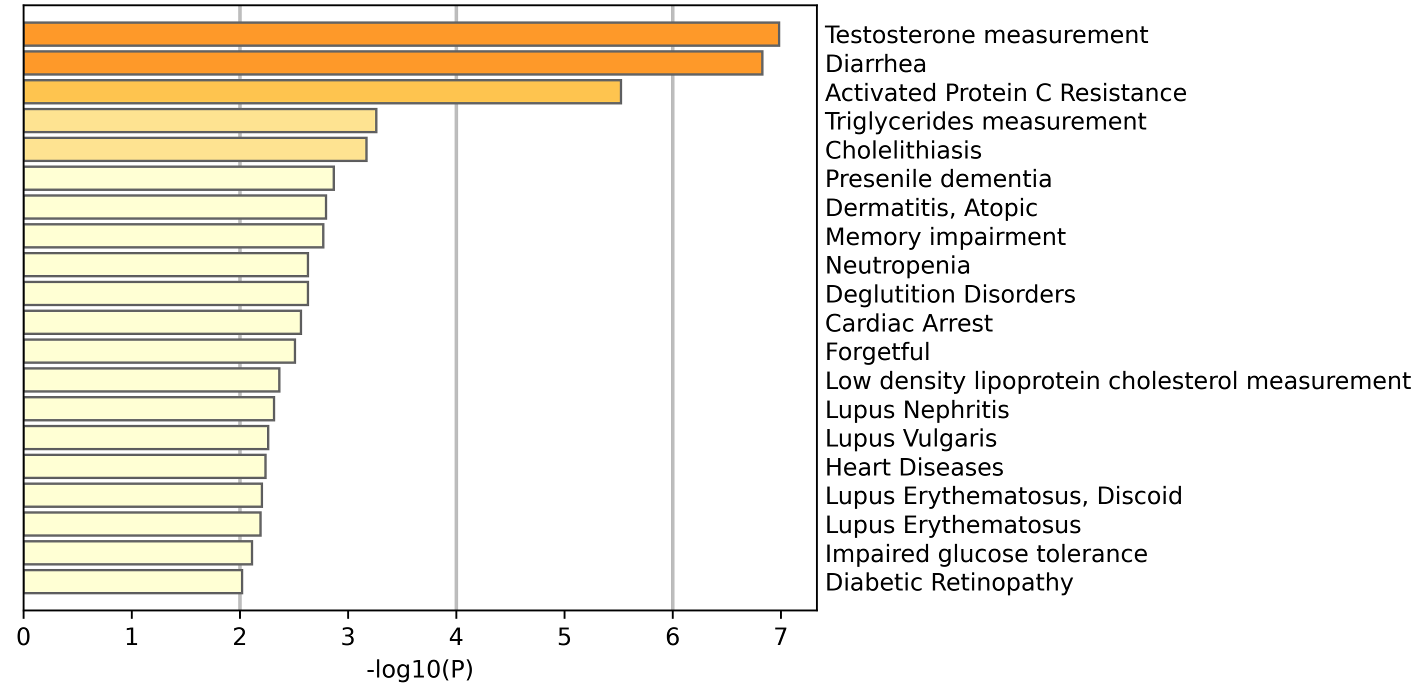

Figure S1: Functional enrichment of 21 proteins

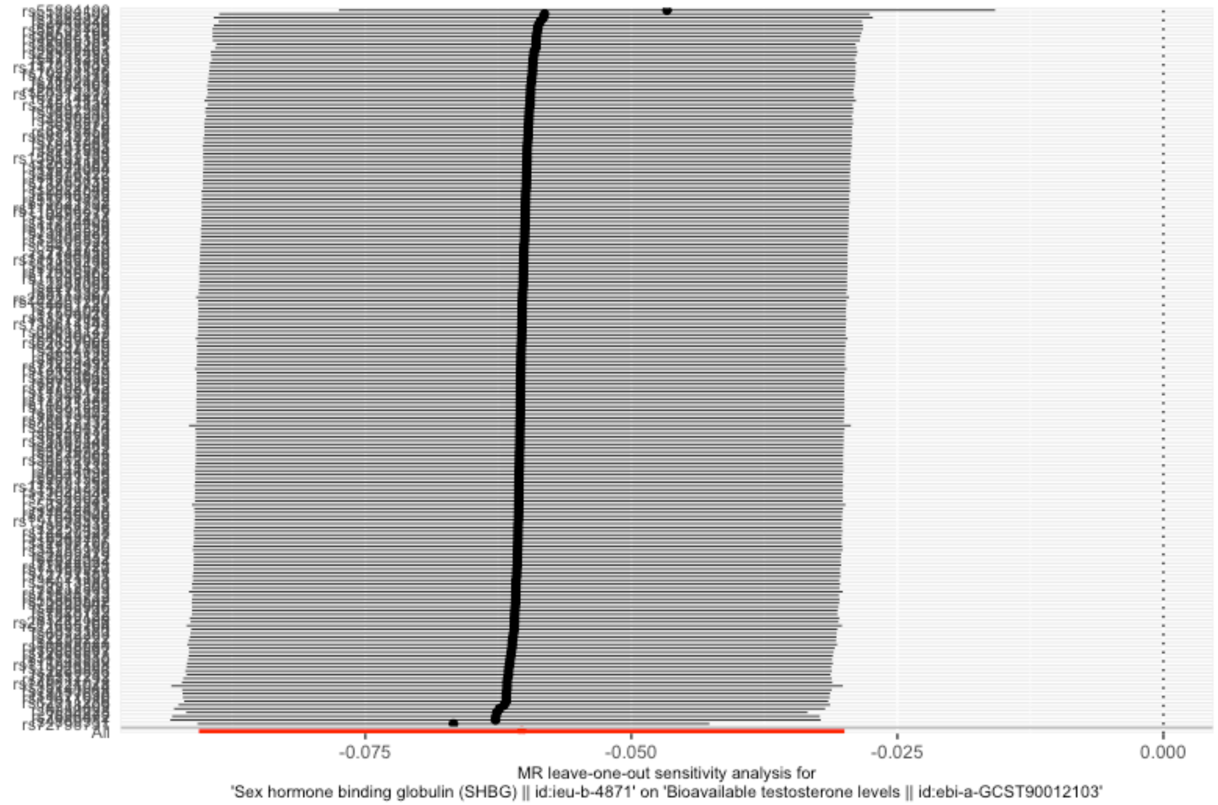

Figure S2: Leave-one-out (SHBG and bioavailable testosterone in male)

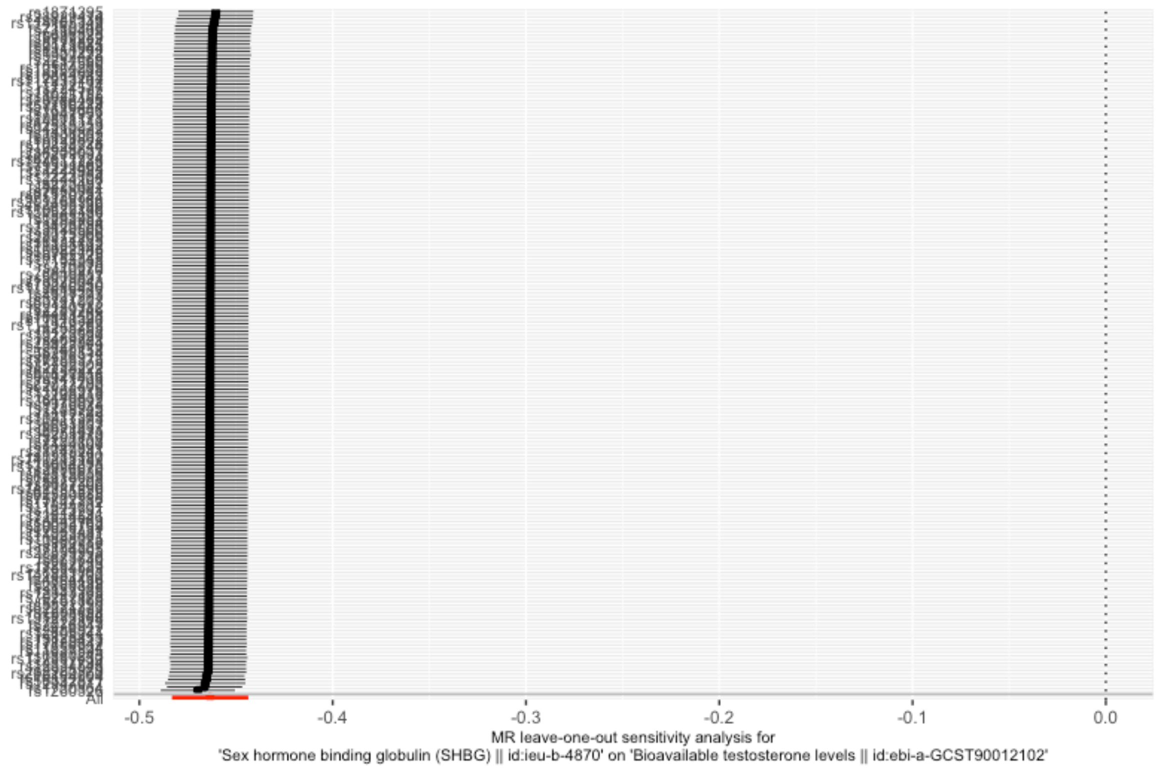

Figure S3: Leave-one-out (SHBG and bioavailable testosterone in female)

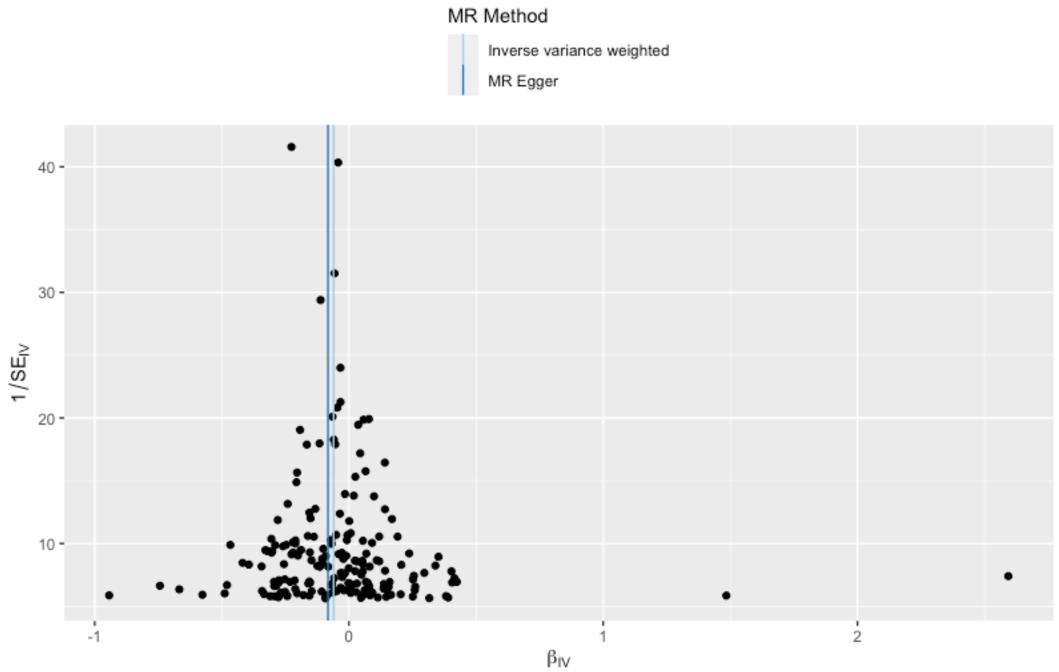

Figure S4: Funnel plot (SHBG and bioavailable testosterone in male)

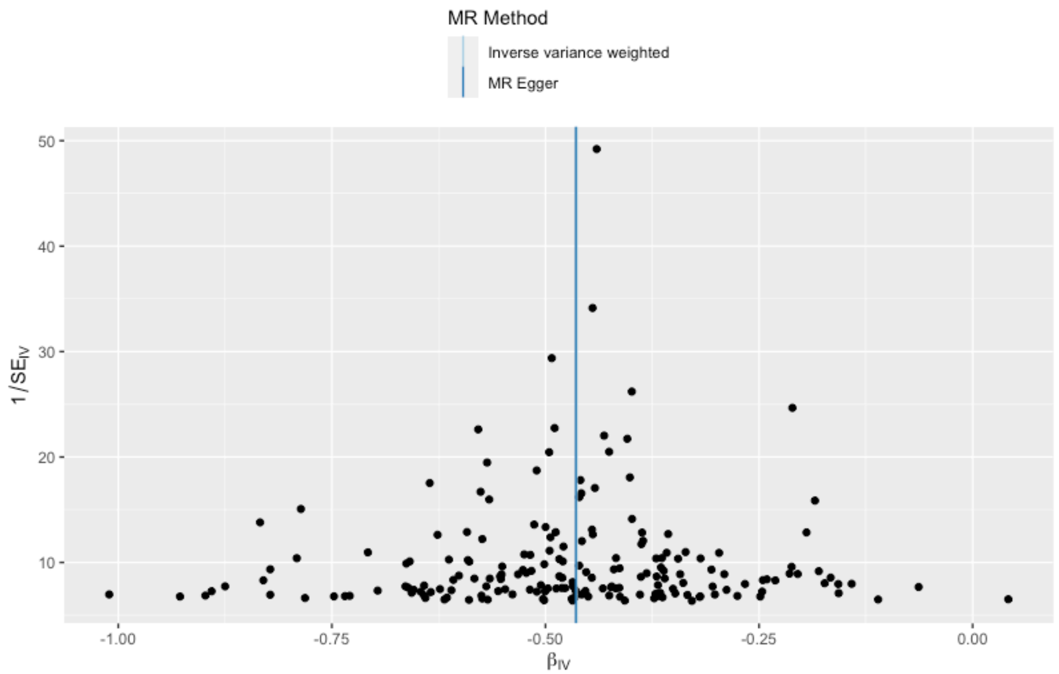

Figure S5: Funnel plot (SHBG and bioavailable testosterone in female)

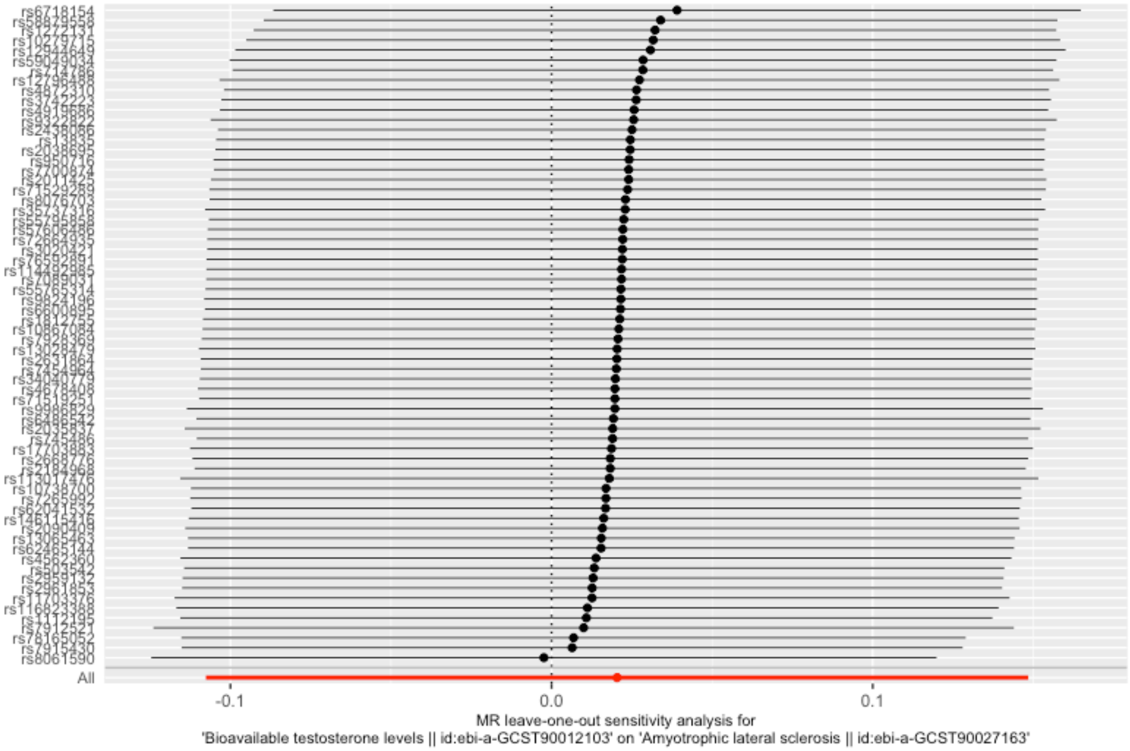

Figure S6: Leave-one-out (Bioavailable testosterone and ALS in male)

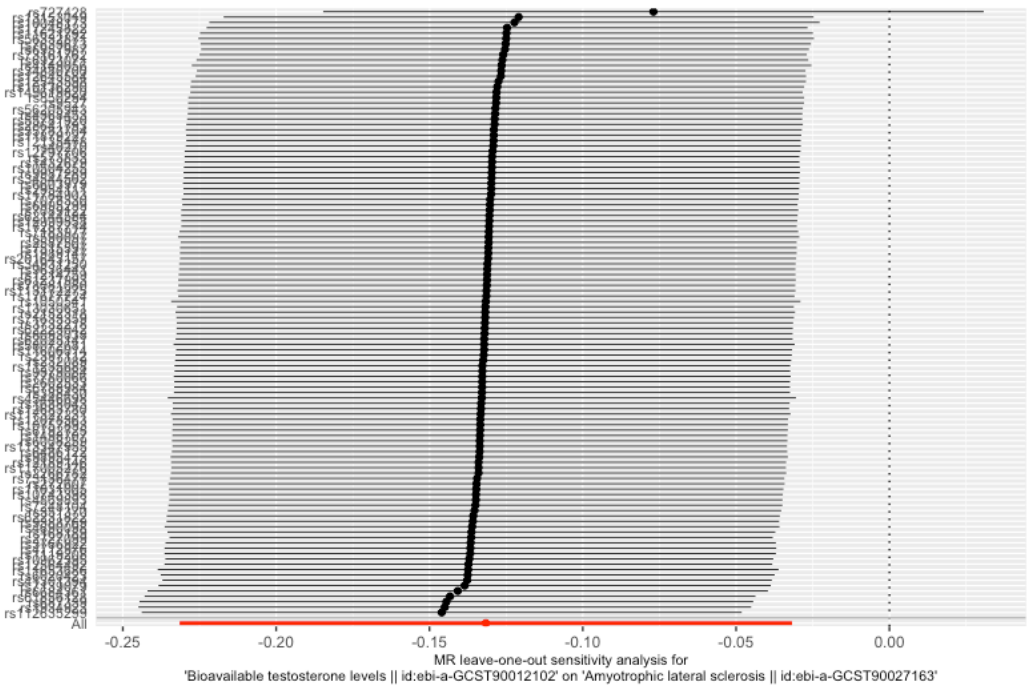

Figure S7: Leave-one-out (Bioavailable testosterone and ALS in female)

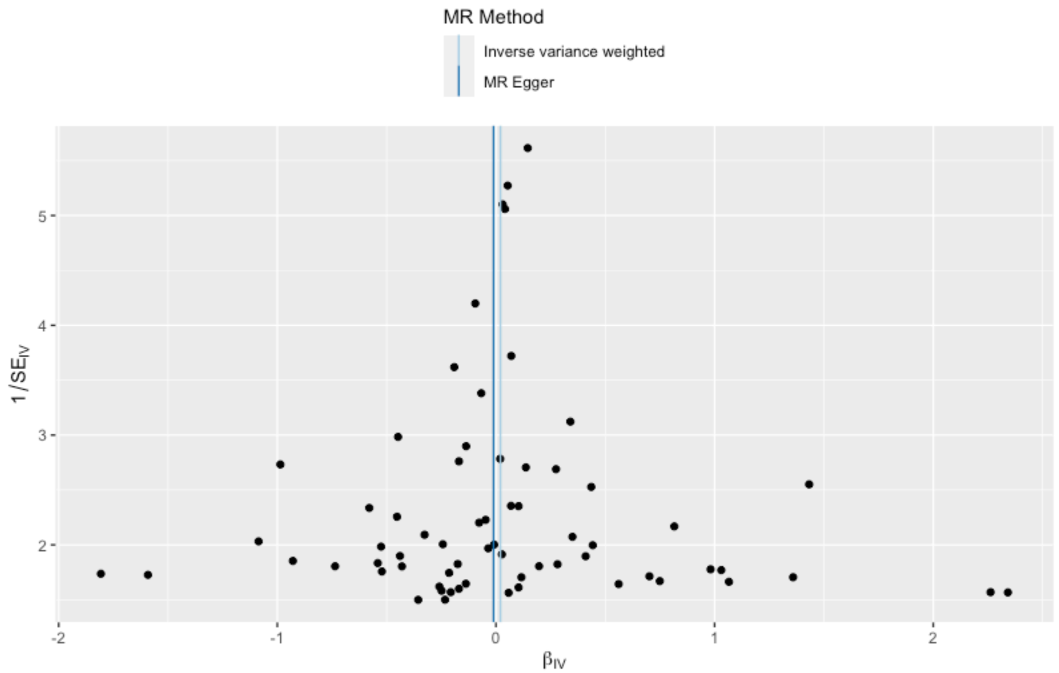

Figure S8: Funnel plot (Bioavailable testosterone and ALS in male)

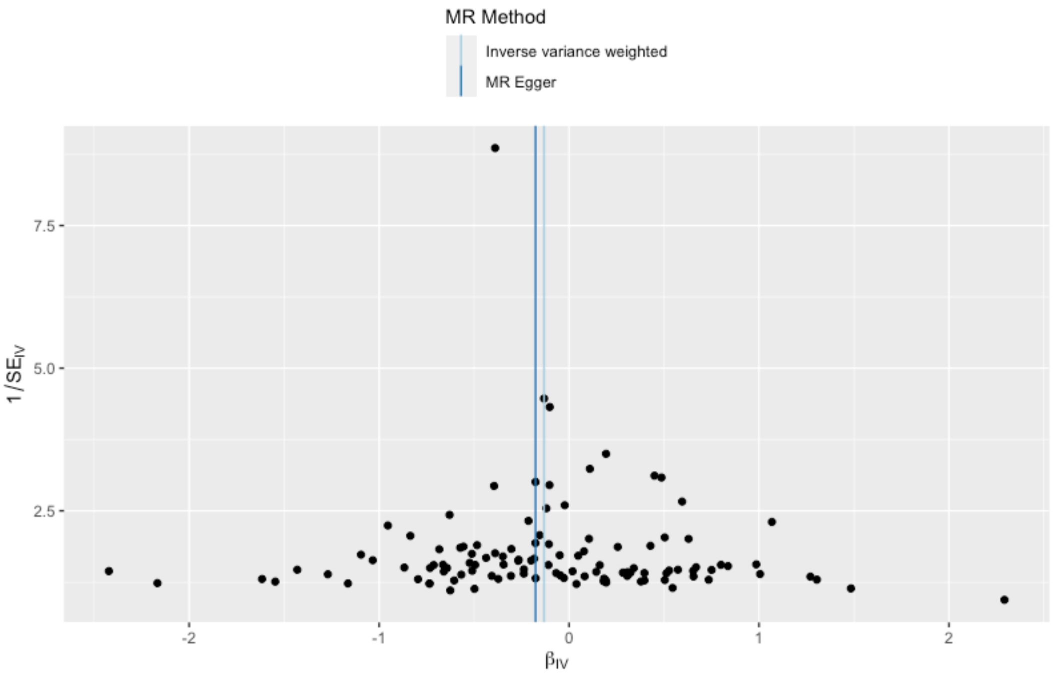

Figure S9: Funnel plot (Bioavailable testosterone and ALS in female)
